# PET/MRI insert using digital SiPMs: Investigation of MR-compatibility

**DOI:** 10.1016/j.nima.2013.08.077

**Published:** 2014-01-11

**Authors:** Jakob Wehner, Bjoern Weissler, Peter Dueppenbecker, Pierre Gebhardt, David Schug, Walter Ruetten, Fabian Kiessling, Volkmar Schulz

**Affiliations:** a Physics of Molecular Imaging Systems, Department for Experimental Molecular Imaging, RWTH Aachen University, Aachen, Germany; b Philips Research Europe, Aachen, Germany; c King's College London, London, United Kingdom; d Philips Research Europe, Eindhoven, The Netherlands; e Department for Experimental Molecular Imaging, RWTH Aachen University, Aachen, Germany

**Keywords:** PET/MRI, Digital SiPM, MR-compatibility

## Abstract

In this work, we present an initial MR-compatibility study performed with the world's first preclinical PET/MR insert based on fully digital silicon photo multipliers (dSiPM). The PET insert allows simultaneous data acquisition of both imaging modalities and thus enables the true potential of hybrid PET/MRI. Since the PET insert has the potential to interfere with all of the MRI's subsystems (strong magnet, gradients system, radio frequency (RF) system) and vice versa, interference studies on both imaging systems are of great importance to ensure an undisturbed operation. As a starting point to understand the interference, we performed signal-to-noise ratio (SNR) measurements as well as dedicated noise scans on the MRI side to characterize the influence of the PET electronics on the MR receive chain. Furthermore, improvements of sub-components’ shielding of the PET system are implemented and tested inside the MRI. To study the influence of the MRI on the PET performance, we conducted highly demanding stress tests with gradient and RF dominated MR sequences. These stress tests unveil a sensitivity of the PET's electronics to gradient switching.

## Highlights

• We successfully operated a fully digital PET insert inside a clinical 3T MRI.
• The main noise source is common mode noise from the PET's power supply.
• Improvements of the power supply's shielding show a significant improvement.
• Stress tests unveil a sensitivity of the PET's electronics to gradient switching.

## 1 Introduction

The combination of Positron Emission Tomography (PET) with its high sensitivity and the possibility for quantitative imaging and Magnetic Resonance Imaging (MRI) has the potential to become the next generation of hybrid imaging techniques [1]. In contrast to the combination of PET with Computed Tomography (CT), MRI offers a better soft tissue contrast and does not use ionizing radiation, thus reducing the overall required radiation dose significantly. To enable the full potential of a hybrid PET/MRI system, both imaging modalities have to work simultaneously, especially to enable a high quality spatial and temporal registration of imaging data at shorter scan times.

An MRI system basically consists of three main components, namely a strong magnet, a gradient system and a radio frequency (RF) system. New detector systems inside the MRI bore, e.g. a preclinical PET insert, have the potential to interfere with all these subsystems of the MRI system and vice versa. Examples for interference phenomena have been reported by several research groups: while Refs. [2], [3], [4] observe image degradation on the MRI side caused by the presence of a PET detector, other groups observe a direct influence on the PET performance caused by the RF pulses of the MRI [5] or switching gradients [6]. The first step to enable the usage of a PET detector inside the MRI was the replacement of photomultiplier tubes with solid state photo detectors, e.g. silicon photomultipliers (SiPM). Especially fully digital Silicon photomultipliers (dSiPMs) offer a good timing, energy and spatial resolution as well as a good temperature stability and they are a promising candidate concerning their MR-compatibility [7], [8]. However, they tend to generate digital electromagnetic noise patterns which might degrade the MR image quality. Thus, proper PET system design and shielding is required to avoid interference. In this work, we started to investigate the interference phenomena and we tested the dSiPMs and our detector architecture concerning MR-compatibility.

## 2 Materials and methods

**Fig. 1:**
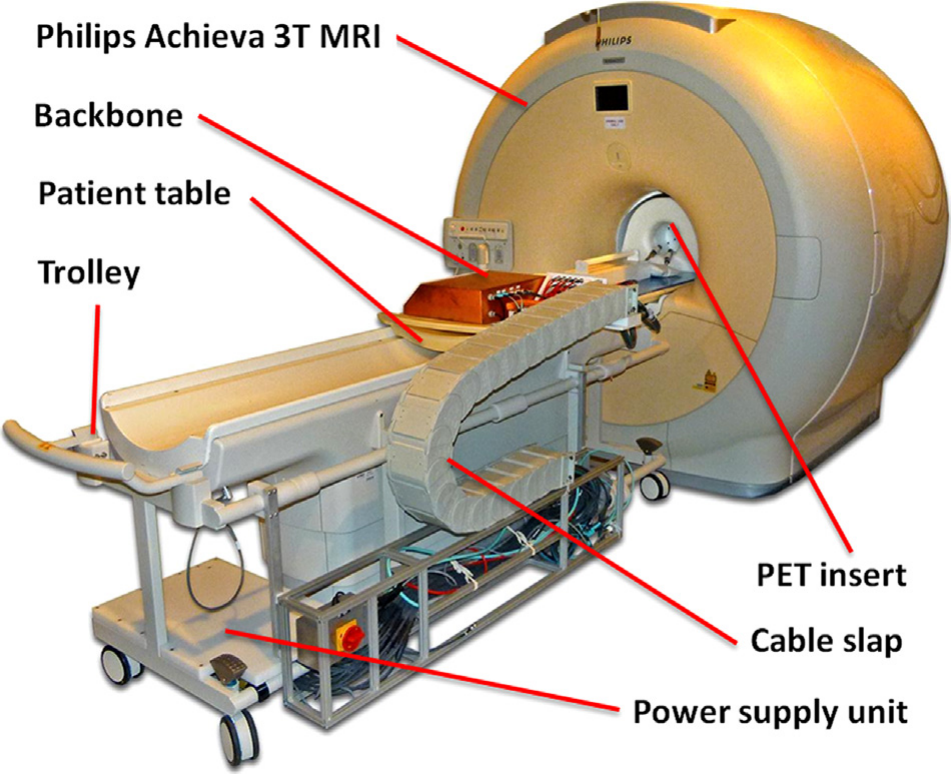
Hyperion-II^D^ PET insert with 10 PET modules (2 stacks each) mounted on the patient table top of a 3T clinical MRI.

**Fig. 2:**
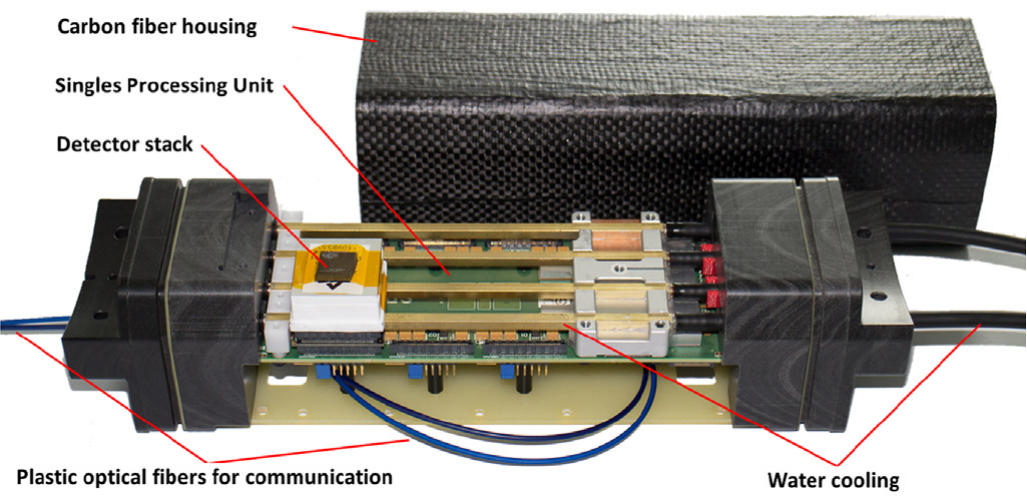
One Singles Detection Module (SDM) hosts up to six detector stacks. Plastic optical fibers are used for synchronization and communication. The module is housed inside a carbon fiber shield.

The Hyperion-II^D^ PET/MR insert and all its support electronics are installed (as shown in Fig. 1) on a patient tabletop and trolley, creating a easy to handle and quickly installable system [9]. The PET ring is made up of ten PET Singles Detection Modules (SDM) which are mounted on a MR-compatible gantry, thus creating a PET ring with diameter of around 210 mm [10]. One SDM (Fig. 2) hosts up to six detector stacks in a 2×3 arrangement (at this stage only two stacks per module are installed) and the communication and synchronization of multiple modules is done via plastic optical fibers (POF) to avoid galvanic connections between the modules and the Data Acquisition and Processing Server (DAPS, similar to the architecture described in [11]) outside the MR examination room. One detector stack is composed of a crystal array (30×30, 1 mm pitch), a 2 mm light guide for light sharing, an 8×8 dSiPM array (DPC 3200-22-44 by Philips Digital Photon Counting) and a local FPGA [7], [8], [12]. The SDM is housed inside an almost gamma transparent carbon fiber screen which shows good RF shielding properties while being mostly transparent for gradients [13]. The insert is designed to fit into a Philips Achieva 3T MRI system and is equipped for MR acquisition with a dedicated PET transparent T/R mouse proton RF-coil (12 leg birdcage, high pass), which has an inner diameter of 46 mm. Consequently, the combined field-of-view (FOV) in this configuration is ∅ 46 mm×33 mm (one of the three possible PET rings installed; up to ∅ 46 mm ×100 mm when all detector stacks are installed).

### 2.1 Influence on the MR performance

To investigate the interference on the MRI system, signal-to-noise ratio (SNR) measurements without and with the PET detector (10 SDMs) are performed. Therefore, a transversal slice of a 50 mm cylindrical phantom (1000 ml demi water, 770 mg CuSO_4_·5 H_2_O, 2000 mg NaCl, 0.05 ml H_2_SO_4_ – 0.1N solution) is imaged using spin echo (SE) sequences (TR/TE: 1000/50 ms, voxel size: 0.25×0.25×1 mm^3^, flip angle: 90°). To study the noise creation by the PET electronics, dedicated noise scans (TSE sequence, TR/TE: 1044/256 ms, TSE factor: 32, acq. matrix: 1024×1024, bandwidth per pixel: 180 Hz) are performed with the complete PET detector and with single PET modules. Experiments with subsystems replacing the complete PET detector are conducted to study the RF interference in detail and to identify the noise's origin. Improvements of the shielding of the power supply unit (PSU) are also realized and tested with these noise scans.

### 2.2 Influence on the PET performance

**Fig. 3:**
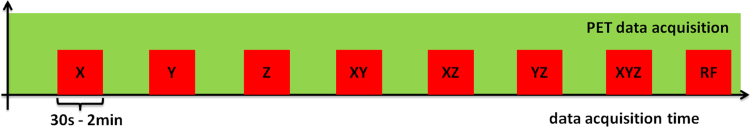
PET data acquisition scheme: PET data is acquired over a longer time period (green) while for certain smaller time windows (30–120 s) RF and gradient stress tests are performed (red). (For interpretation of the references to color in this figure caption, the reader is referred to the web version of this paper.)

The PET performance and stability during simultaneous operation was studied with one PET module (equipped with one stack) by single event detection. PET data and system parameters like voltages, currents and temperatures are acquired using a ^22^Na point source (activity: 2.8 MBq) over a longer time period (several minutes up to 45 min). During this data acquisition (as shown in Fig. 3), highly demanding RF and gradient stress tests (with various switching directions) are performed in smaller time windows (30 s–2 min).

For the gradient tests, EPI sequences (EPI factor: 49) with maximum gradient strength (30 mT), maximal slew rates and minimal TR with defined switching directions (the individual parameters are listed in Table 1) are used and for the RF test a highly demanding TSE sequence (TSE factor: 16, TE/TR: 21/333 ms, peak *B*_1_ amplitude: 20μT) is executed.

**Table 1:** Overview of the slew rates, TR and TE of the used gradient dominated sequences.

Sequence	Slew rate (mT/m/ms)	TR/TE (ms)
X	184.8	28/13
Y	194.5	27/13
Z	192.3	25/12
XY	198.0	27/13
XZ	197.9	26/12
YZ	197.6	24/12
XYZ	198.1	26/12

## 3 Results and discussion

### 3.1 Influence on the MR performance

**Fig. 4:**
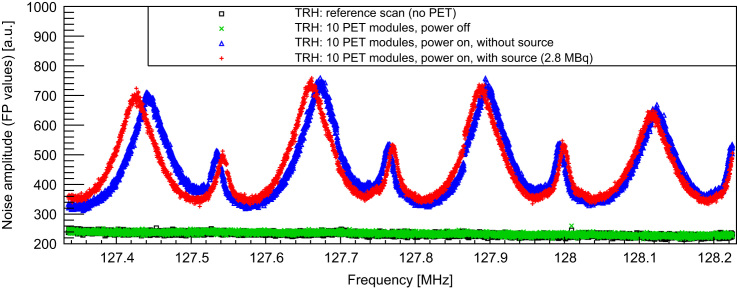
Noise scan results without (black) and with PET detector (green: power off, blue: power on, red: power on + source (2.8 MBq)). The noise floor is strongly increased and shows broad peaks which are 250 kHz apart, corresponding to the switching frequency of the switched mode power supply. (For interpretation of the references to color in this figure caption, the reader is referred to the web version of this paper.)

**Fig. 5:**
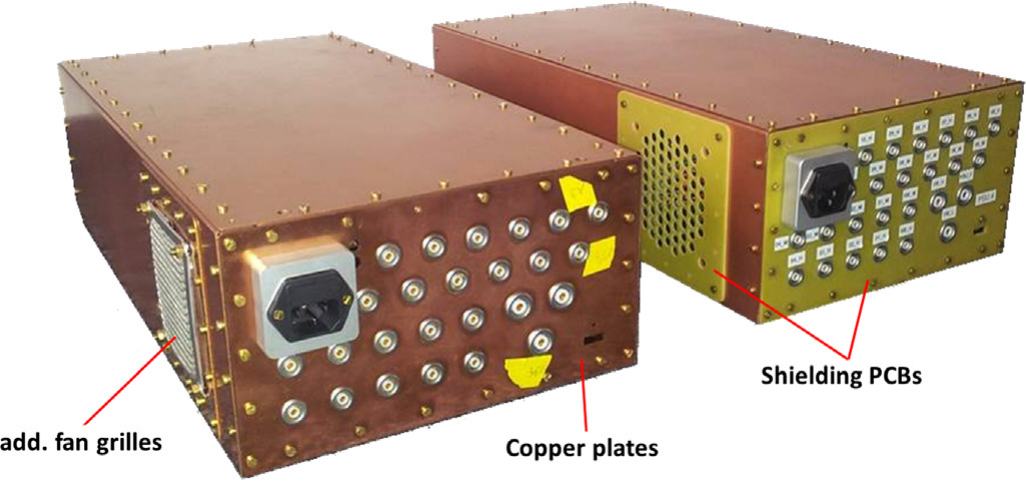
Comparison of the switched mode power supplies (right: unmodified PSU, left: improved version): the shielding of the PSU is improved by replacing all shielding PCBs (yellow) by thick copper plates. Additional fan grilles are installed on the outside to reduce the leaking EM fields. (For interpretation of the references to color in this figure caption, the reader is referred to the web version of this paper.)

**Fig. 6:**
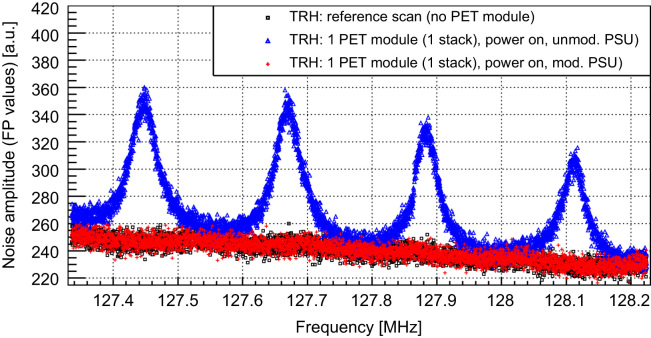
Noise scans without PET (reference, black) and with PET (blue: unmodified PSU, red: modified PSU). Measurements were performed with one PET module (equipped with one stack) and the dedicated mouse coil. (For interpretation of the references to color in this figure caption, the reader is referred to the web version of this paper.)

The SNR study shows a strong influence of the PET system on the MR performance: while the reference scan without PET detector delivers an MR image with a SNR (calculated according to NEMA standard [14]) of 159, the measurement with the PET detector (10 SDMs, power on, data acquisition) reveals a SNR degradation by a factor of 2 (SNR: 81) [9]. Fig. 4 shows the corresponding noise spectrum of the RF system without PET detector (black) and with detector (green: powered off and unplugged from the AC outlet, blue: data acq., red: data acq. + 2.8 MBq ^22^Na source). The noise floor is strongly increased, when the PET detector is switched on (in the frequency range of the SE images by a factor of approx. 1.9, thus explaining the observed SNR degradation), and features broad peaks which are shifting as a function of time, especially in the heat up phase of the scanner. These peaks are approximately 250 kHz apart, which is also the switching frequency of the last converter in the employed switched mode PSU. Experiments with purely resistive loads replacing the PET modules have identified clearly common mode noise originating from the PSU as main noise source. As a consequence, we improved the PSU's shielding as shown in Fig. 5 (left: improved version, right: unmodified PSU): all shielding PCBs are replaced by thick copper plates, cables near the fan grilles and the cooling fans themselves are removed to avoid field leaking through the fan grilles, and we installed additional fan grilles on the outside as a second chamber, to reduce the leaking electromagnetic (EM) fields. A drawback of this solution is the creation of a second DC star point: the shields of the power cables (one SDM is provided by three power cables) are connected together on the PSU side and module side, thus creating potentially problematic loops in which switching gradients and RF pulses from the MRI could couple in and could cause trouble with the supply of the SDMs. Furthermore, an effective shielding of the coaxial cables cannot be guaranteed anymore. Based on this restrictions, the presented solution might be seen as intermediate step towards a final solution. Fig. 6 shows the resulting noise scans of one SDM (equipped with one Stack) with the different PSU versions. No obvious difference between the reference noise floor (black, without PET, average noise floor (floating point values (FPV)): 239.7 (STDV: 7.9)) and the one with the modified PSU (red, average noise floor (FPV): 239.9 (STDV: 7.8)) can be observed. In comparison to the unmodified PSU, the modifications show clearly an improvement of the noise situation and so far, the drawbacks of the modifications have not harmed the operation of one single SDM.

**Fig. 7:**
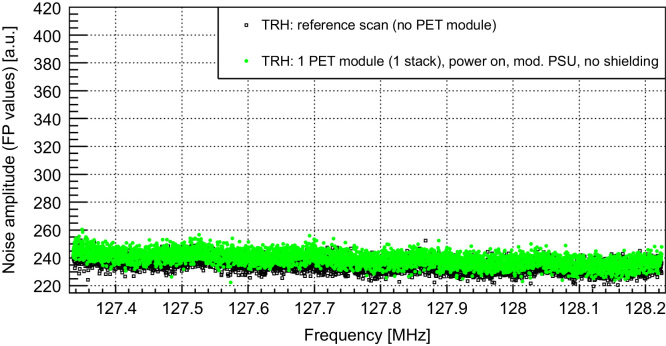
Noise scans without PET (reference, black) and with PET (green, without carbon RF shielding). Measurements were performed with one PET module and the dedicated mouse coil. (For interpretation of the references to color in this figure caption, the reader is referred to the web version of this paper.)

On top of that, measurements without carbon fiber RF shielding (Fig. 7, green, average noise floor (FPV): 239.5 (STDV: 5.0)) show only a slight increase in the noise floor compared to the reference scan (black, average noise floor (FPV): 235.3 (STDV: 4.6)). This increase appears to be negligible compared to the noise produced by the PSU. The noise scan does not show any digital noise which manifests itself as sharp spikes in the scan: the emitted digital noise seems to fall into a frequency range in which the MRI acquisition chain is insensitive and thus does not appear in the MRI noise scans. It is to note at this point that these measurements are performed with one single SDM equipped with one stack. A fully equipped PET ring consists of 10 SDMs containing 60 detector stacks and has the potential to disturb the acquisition of the MRI much more.

### 3.2 Influence on the PET performance

**Fig. 8:**
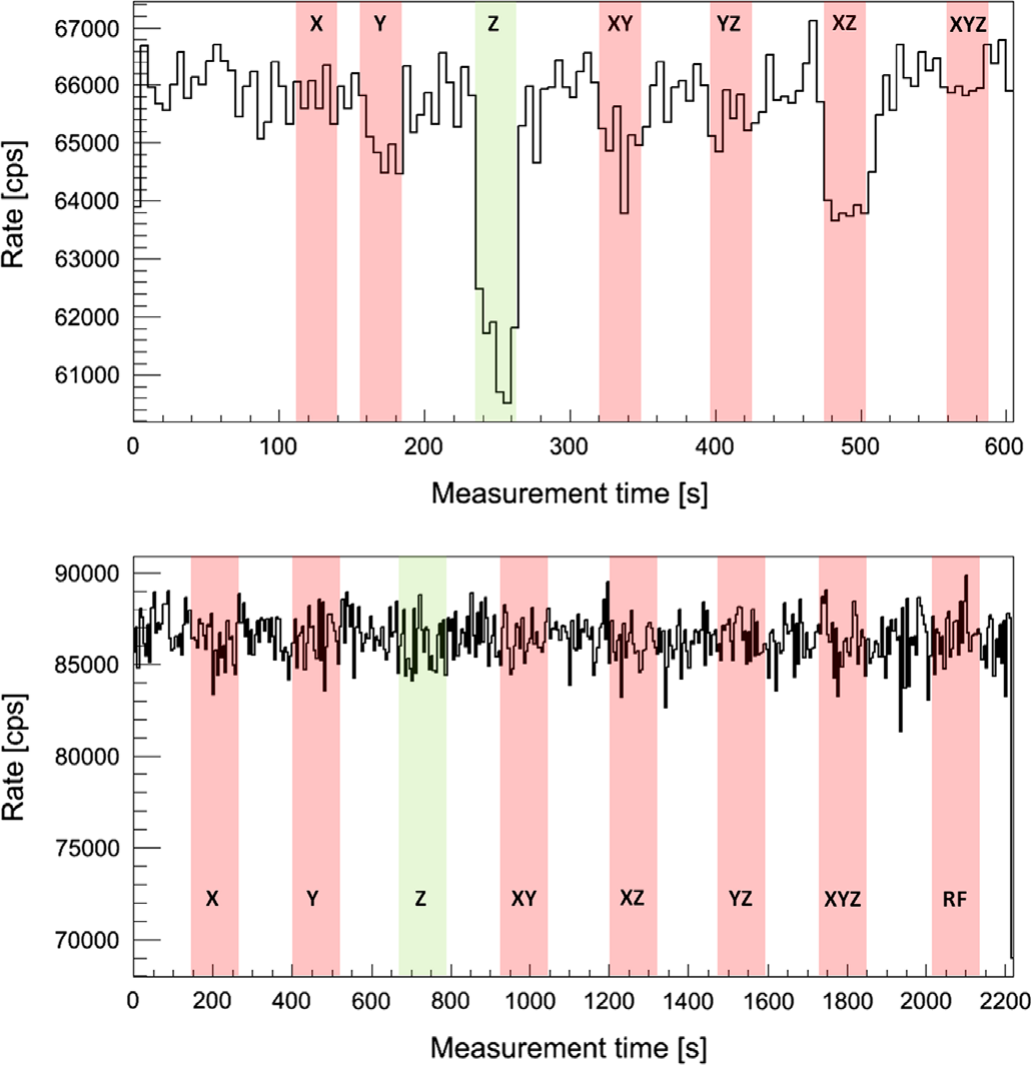
Singles rate (in counts per second (cps), averaged over 5 s intervals, energy cut: (511±30) keV, all pixel around main pixel present) during highly demanding gradient sequences with different switching directions (as indicated with color shaded areas) for two different over voltages (OV) (top: OV=2.9 V, bottom: OV=2.5 V). Only the measurement with the higher OV shows singles rate drops by up to 5.6% during active z gradient sequences. (For interpretation of the references to color in this figure caption, the reader is referred to the web version of this paper.)

**Fig. 9:**
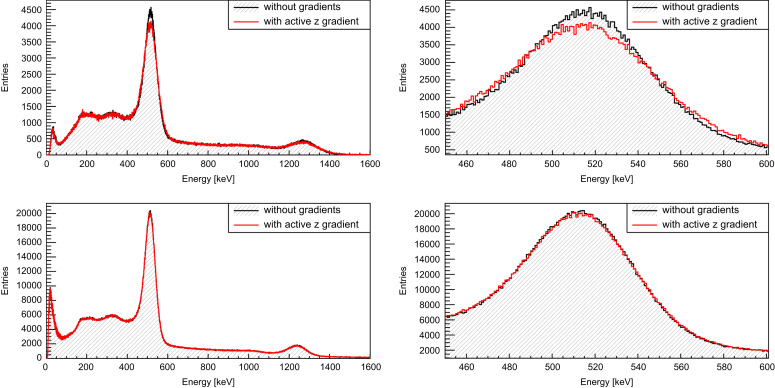
Energy histogram (left: overall, right: photo peak range) for time windows without gradient switching (black, shaded) and with switching z gradients (red) for two different over voltages (OV) (top row: OV=2.9 V, bottom row: OV=2.5 V). (For interpretation of the references to color in this figure caption, the reader is referred to the web version of this paper.)

**Fig. 10:**
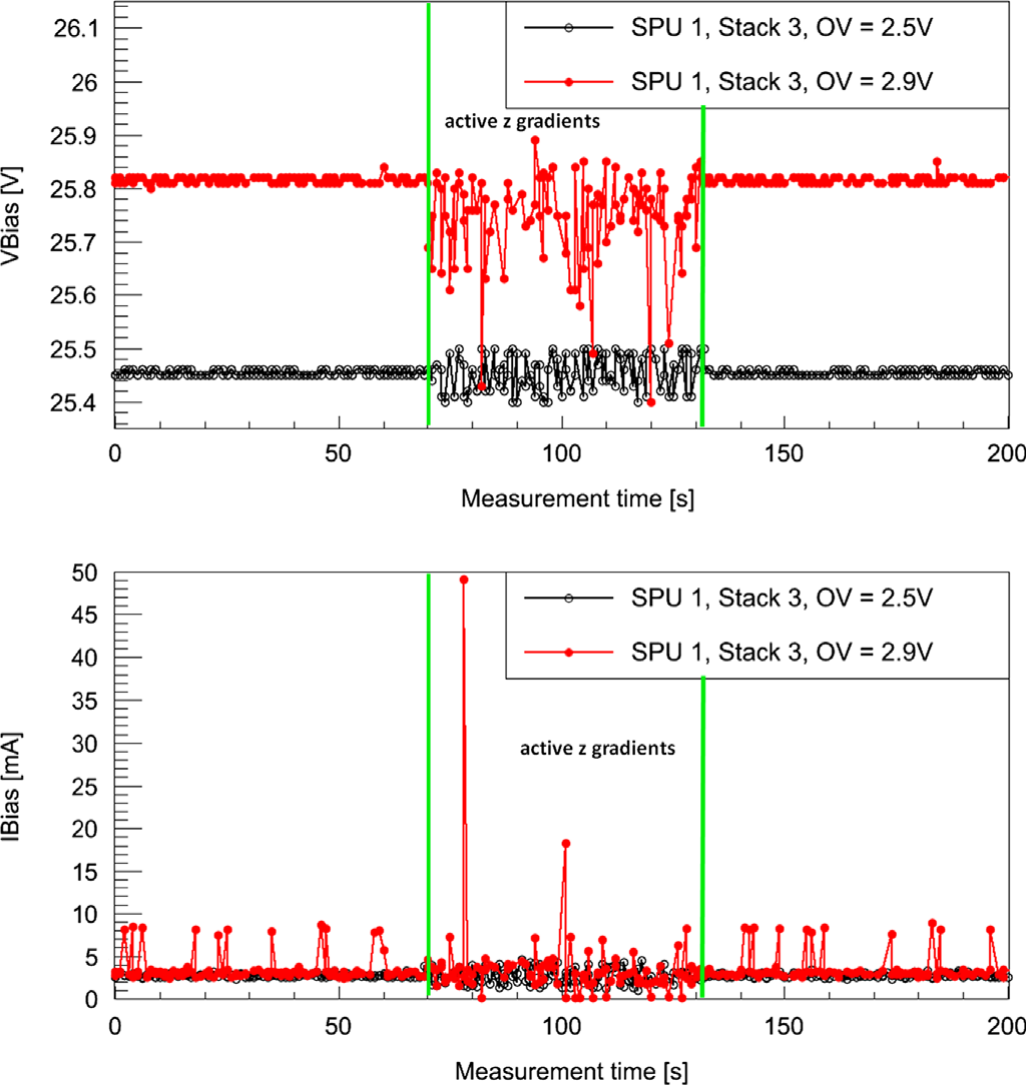
Measurement of the bias voltage *V*_*B*_ (top) and bias current *I*_*B*_ (bottom) for two different over voltages (OV) (black: OV=2.5 V, red: OV=2.9 V) as a function of time. In time regions with active z gradients (indicated by green boundaries) a strong ripple on *V*_*B*_ and *I*_*B*_ occurs. (For interpretation of the references to color in this figure caption, the reader is referred to the web version of this paper.)

A first evaluation of acquired PET data inside and outside the MRI shows no degradation of PET data quality and PET performance: for instance, flood histograms and energy resolution remain unaffected [12]. The singles data rates of the PET modules are stable even during harsh MRI sequences [9]. However, the stress tests as described in 2.2 reveal sensitivity for gradient switching. Fig. 8 shows the singles rate (for one sensor tile, quality requirements: energy cut ((511±30) keV), all neighboring pixels around the main pixel present (quality cut on the light distribution)) for different bias voltages *V*_*B*_ (upper row: *V*_*B*_=25.8 V, over voltage (OV) =2.9 V ; bottom row: *V*_*B*_=25.4 V, OV =2.5 V) as a function of time. The time windows with the corresponding gradient switching directions are indicated by color (red and green) shaded areas. While the measurement with the OV of 2.9 V shows singles rate drops in time regions with active z gradients by about 5.6%, the measurement with the lower bias voltage shows a stable data acquisition without any rate drops. Interestingly, the measurement with the higher OV (2.9 V) shows a lower count rate than the one with the lower OV indicating that the higher OV is not good operating point. This hypothesis is supported by a worse energy resolution for the higher OV (energy resolution without gradient switching: 15.3% for OV=2.9 V and 12.9% for OV=2.5V). Fig. 9 shows the corresponding singles energy spectra (same quality cuts as before except energy cut) in time windows without gradients (black, shaded) and with active z gradient (red). For the measurement with 2.9 V OV (top row; left: overall energy spectrum, right: photo peak range), we observe a broadening effect of the energy resolution by 2% (without gradients: 15.3%; with gradients: 17.3%) which causes, after applying the energy cut, the described singles rate drop of 5.6%. Furthermore, the singles count (integral over the entire energy spectrum) with active gradients *N*_*AZ*_ is in comparison with the number of singles without gradient switching *N*_*NZ*_ slightly reduced ($\Delta N=N_{\mathrm{NZ}}-N_{\mathrm{AZ}}=6559\pm 1461$ (ratio: $N_{\mathrm{AZ}}/N_{\mathrm{NZ}}=0.994$)). In contrast to this finding, the measurements with low *V*_*B*_ (bottom row; left: overall energy spectrum, right: photo peak range) show no broadening effect (energy resolution: 12.9%) and thus no drops in the singles rate. The overall count of singles is not significantly affected by the presence of switching gradients ($\Delta N=N_{\mathrm{NZ}}-N_{\mathrm{AZ}}=2155\pm 3089$ (ratio: $N_{\mathrm{AZ}}/N_{\mathrm{NZ}}=0.9995$)). Measurements of *V*_*B*_ (top) and bias current *I*_*B*_ (bottom) during data acquisition without and with active z gradients (black: OV =2.5 V, red: OV =2.9 V) are shown in Fig. 10: the switching z gradients generate obviously a ripple on *V*_*B*_ and *I*_*B*_ which is especially dominant for an OV of 2.9 V. It seems that this *V*_*B*_ setting is chosen too high since *I*_*B*_ fluctuates strongly and uncontrollably. One explanation for this strong response on the gradient induced *V*_*B*_ fluctuations might be that the active quenching mechanism does not work properly anymore. This would also be an explanation for the observed degradation of the energy resolution (which is only present at the higher OV setting) since the reload mechanism of the micro cells is disturbed, leading to an additional spread of the photon count values. It is to note that the origin of the *V*_*B*_ fluctuations and the exact influence of these fluctuations on the detector performance is unclear at this stage. The above mentioned explanation is currently a working theory and has to be proven by further investigations.

## 4 Conclusion

We have successfully operated a fully digital PET detector inside a 3T MRI. On the MRI side, we observed a SNR degradation by a factor of 2 which is mainly caused by common mode noise from the switched mode power supply. Improvements on the PSU's shielding lead to a notable reduction of the noise. In the latest modification no difference between the reference noise floor and the one during PET acquisition is visible. Although we observed no technical problems with the modifications during the operation with one SDM, a final evaluation with a complete PET scanner has to be done. Until this test is conducted, the presented solution can only be seen as intermediate step.

On the PET side, we notice that our PET system works stable even under unrealistic demanding stress tests. Up to now, we do not observe any hiccups in the data communication. However, the performed stress tests reveal a sensitivity for switching z gradients. We observe a ripple on the bias voltage and a broadening effect of the energy resolution for an aggressive chosen bias voltage setting. After application of a narrow energy cut ((511±30) keV) around the photo peak, this broadening effect leads to a singles rate drop by approx. 5.6%. The coupling mechanism of the gradients causing the *V*_*B*_ fluctuations as well the influence of these fluctuations on the energy resolution is unclear at this stage. These questions will be subject of further investigations.
